# Renal parenchymal volume analysis: Clinical and research applications

**DOI:** 10.1002/bco2.70013

**Published:** 2025-03-19

**Authors:** Carlos Munoz‐Lopez, Kieran Lewis, Nityam Rathi, Eran Maina, Akira Kazama, Anne Wong, Angelica Bartholomew, Worapat Attawettayanon, Yunlin Ye, Zhiling Zhang, Wen Dong, Rebecca A. Campbell, Nicholas Heller, Erick Remer, Christopher Weight, Steven C. Campbell

**Affiliations:** ^1^ Glickman Urological and Kidney Institute, Cleveland Clinic Cleveland OH USA; ^2^ Department of Urology, Division of Molecular Oncology, Graduate School of Medical and Dental Sciences Niigata University Japan; ^3^ Division of Urology, Department of Surgery, Faculty of Medicine, Songklanagarind Hospital Prince of Songkla University Songkhla Thailand; ^4^ Department of Urology, Sun Yat‐sen University Cancer Center Guangzhou P. R. China

**Keywords:** functional recovery, kidney cancer, new baseline glomerular filtration rate, parenchymal volume analysis, partial nephrectomy, radical nephrectomy, split renal function

## Abstract

**Background and Objectives:**

In most patients, the renal parenchymal volumes in each kidney directly correlate with function and can be used as a proxy to determine split renal function (SRF). This simple principle forms the basis for parenchymal volume analysis (PVA) with semiautomated software, which can be leveraged to predict SRF and new‐baseline glomerular filtration rate (NBGFR) following nephrectomy. PVA was originally used to evaluate renal transplantation donors and has replaced nuclear renal scans (NRS) in this domain. PVA has subsequently been explored for the management of patients with kidney cancer for whom difficult decisions about radical versus partial nephrectomy can be influenced by accurate prediction of NBGFR. Our objective is to present a comprehensive review of the applications of PVA in urology including their clinical and research implications.

**Methods:**

Key articles utilizing renal PVA to improve clinical care and facilitate urologic research were reviewed with special emphasis on take‐home points of clinical relevance and their contributions to progress in the field.

**Results:**

There have been considerable advances in renal PVA over the past 15 years, which is now established as a reference standard for the prediction of functional outcomes after renal surgery. PVA provides improved accuracy when compared to NRS‐based estimates or non‐SRF‐based algorithms. PVA can be performed in minutes using routine preoperative cross‐sectional imaging and can be readily applied at the point of care. Additionally, PVA has important research applications, enabling the precise study of the determinants of functional recovery after partial nephrectomy, which can affect surgical approaches to this procedure.

**Conclusions:**

Despite the wide availability of PVA, primarily for use in renal transplantation, it has not been widely implemented for other urologic purposes at most centres. Our hope is that this narrative review will increase PVA utilization in urology and facilitate further progress in the field.

## 1 INTRODUCTION

Functional outcomes after partial nephrectomy (PN) or radical nephrectomy (RN) are important elements of cancer survivorship for patients with localized kidney cancer.^1^, ^2^, ^3^, ^4^ The ability to predict new‐baseline GFR (NBGFR) after surgery has undergone substantial evolution over the past two decades. Prior generations of urologists relied on subjective assessments for “prediction” of NBGFR based on the evaluation of each kidney relative to the other, considering the degree of enhancement and size. Current AUA guidelines emphasize the prediction of NBGFR for the selection of RN vs PN in complex situations, and there have been significant efforts to develop nomograms that accurately predict NBGFR after renal surgery.^1^ The most accurate of these models leverages preoperative SRF, which was historically derived through nuclear‐renal‐scans (NRS), and renal‐functional‐compensation (RFC) to estimate NBGFR.^4^, ^5^, ^6^ However, estimates from NRS suffer from a high degree of inter‐ and intra‐observer variability due to subjectivity associated with defining the area of interest for measuring the functional activity of each kidney.^7^

In the past decade, a new methodology has been introduced that can more accurately estimate SRF in a user‐friendly, inexpensive and highly reproducible manner. This software only requires cross‐sectional imaging to measure the relative parenchymal volumes on each side. This approach, termed differential parenchymal‐volume‐analysis (PVA), has substantially improved our prediction and analysis of functional outcomes after RN/PN, allowing for more refined patient counselling/management and facilitating research progress. Despite these advances and the availability of PVA software at most centres, primarily for use in renal transplantation, PVA has not been widely implemented in clinical practice for renal cancer patients. Our objective is to highlight the technological advances in PVA over the past decade with a comprehensive discussion of how PVA has advanced clinical care and research for renal cancer.

## 2 CLINICAL APPLICATIONS

### 2.1 Clinical significance of NBGFR

After renal cancer surgery, the remaining parenchyma requires several days to establish its NBGFR, after which the GFR tends to remain stable other than modest age‐related long‐term changes.^4^, ^8^ After the typical clamped PN, the ipsilateral kidney must recover from ischemia, and after RN, RFC of 20–30% typically occurs in the contralateral kidney to yield the NBGFR.^4^, ^9^ The importance of NBGFR is apparent in multiple scenarios, including treatment decision‐making in kidney cancer and upper tract urothelial carcinoma (UTUC), as well as for assessment of living‐donor transplant eligibility.

Decisions about PN vs. RN can be challenging with careful consideration of functional and oncologic issues and risks of perioperative morbidity, which can be impacted by the surgical approach in select patients.^1^, ^4^ While PN is generally preferred due to improved functional outcomes, RN is occasionally required. Current AUA guidelines recommend considering RN whenever there is increased oncologic potential based on tumour size, preoperative renal‐mass‐biopsy or locally‐advanced imaging features. In this setting, RN is preferred if all of the following criteria are also met: 1) high tumour‐complexity; 2) absence of preexisting chronic kidney disease (CKD); and 3) presence of a normal contralateral kidney that will likely provide NBGFR>45 ml/min/1.73m^2^ even if RN is performed.^1^ The latter requirement is based on data demonstrating increased CKD‐related mortality in patients with a NBGFR<45 ml/min/1.73m^2^ after renal cancer surgery.^2^, ^3^, ^8^ In contrast, patients with NBGFR>45 ml/min/1.73m^2^ have renal stability and overall survival comparable to patients without CKD.^1^, ^3^

Accurate prediction of NBGFR is also important for patients with UTUC because NBGFR following radical nephroureterectomy (RNU) may influence the timing of systemic platinum‐based chemotherapy. AUA guidelines recommend neoadjuvant platinum‐based chemotherapy for patients whose NBGFR is expected to be <60 ml/min/1.73m^2^ following RNU.^10^ However, most clinicians view this as overly conservative and are willing to treat patients with GFR > 45 ml/min/1.73m^2^, but not below this threshold.^11^ Patients predicted to have an acceptable NBGFR post‐RNU can have surgery upfront and then receive adjuvant platinum‐based chemotherapy only if the pathology confirms locally advanced disease.^10^

For renal transplantation, comprehensive preoperative assessment of donor renal function is essential.^12^ Compared to kidney cancer and UTUC patients, most living donors tend to be younger/healthier, with better renal function. Careful preoperative evaluation is required to ensure that the donor retains the better kidney and that the donated kidney will provide adequate function after transplantation.

### 2.2 Importance of SRF

Numerous approaches have been developed to predict NBGFR following renal surgery (Supplemental Table S1). These methods have variable accuracy and many are complex, incorporating GFR, age and comorbidities with each weighted to reflect relative impact.^4^, ^13^, ^14^ A simpler approach is to prioritize the relative contributions of each kidney to global GFR, known as SRF, and multiple studies have demonstrated the superiority of SRF‐based models when compared to non‐SRF‐based methods.^5^, ^6^ For patients being considered for nephrectomy, only the GFR contribution from the remaining (contralateral) kidney is relevant for NBGFR prediction. Following nephrectomy, the contralateral kidney will undergo RFC of approximately 20%–30% in the average adult.^9^, ^15^ Therefore, the NBGFR following total nephrectomy can be approximated by: 1.25(Global GFR_Pre‐RN_)(SRF_Contralateral_). The benefits of this SRF‐based methodology include its accuracy and simplicity because it only requires preoperative GFR and SRF.

Historically, SRF has been determined by NRS, which utilizes radiolabeled tracers like Tc‐99 m‐mercaptoacetyltriglycine (MAG3) to capture the relative uptake and excretion of tracer from each kidney. For decades, MAG3 scans were the preferred method for SRF estimation in both living donor kidney transplants and preoperative kidney cancer evaluation, despite their inaccuracy.^7^ The landscape of SRF estimation has undergone rapid evolution, as new methodologies have been developed utilizing relative renal size to estimate SRF. In most patients, differential renal parenchymal volumes directly correlate with function and can be used as an accurate proxy to determine SRF. This simple principle forms the basis for PVA, and how it can be leveraged to predict SRF and NBGFR and improve patient management.

### 2.3 Methods for PVA

#### 2.3.1 Linear renal measurement

**FIGURE 1:**
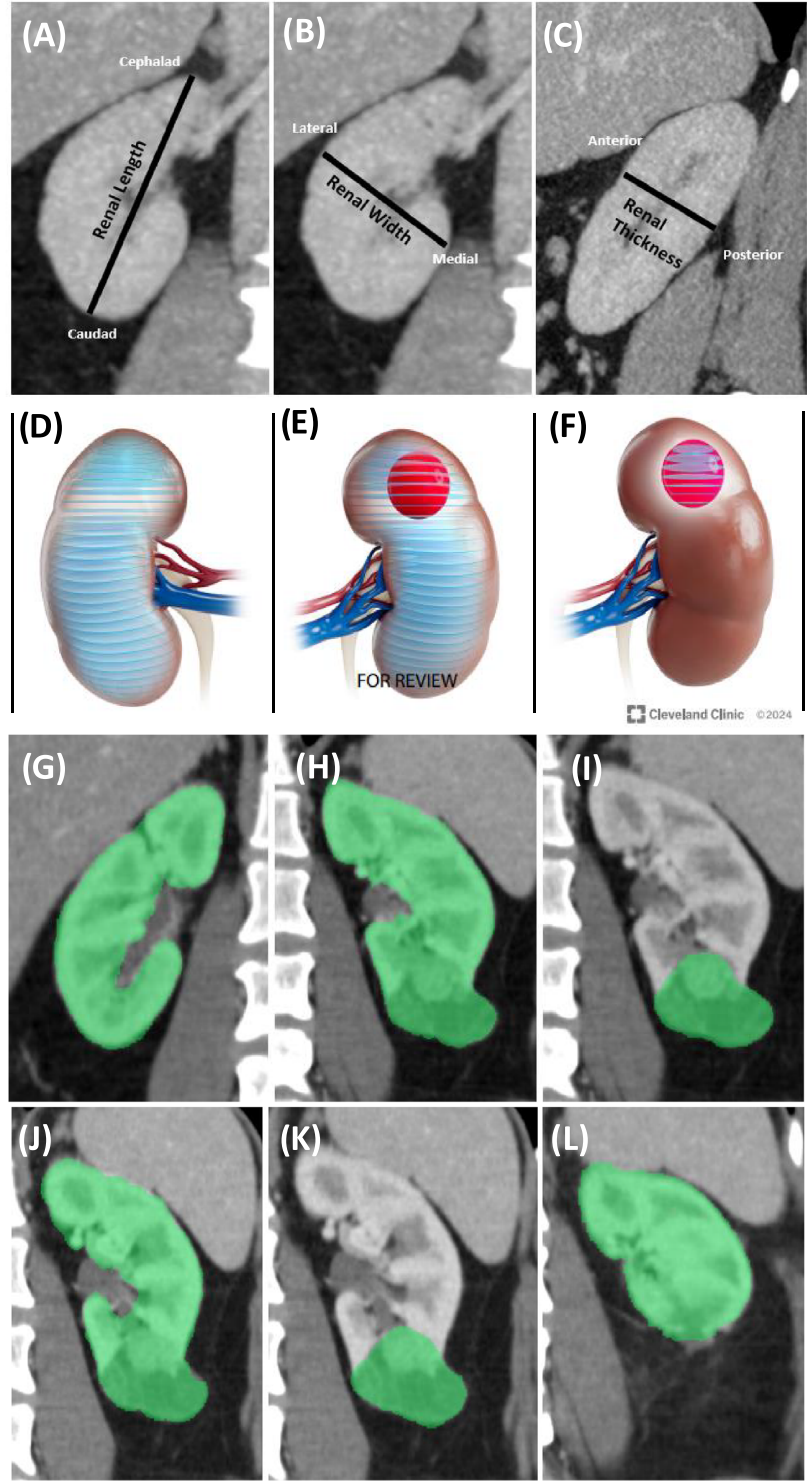
Various approaches to perform parenchymal volume analysis to obtain renal functional metrics, such as split renal function (SRF) and percent parenchymal volume preserved (PPVP). (A–C) Estimation of renal parenchymal volume based on linear measurements of the length, width and height (LWH) as described by Schober et al.^16^ and Feder et al.^17^ (A) Linear LWH measurements showing length (cephalad to caudad, coronal view); (B) width (lateral to medial, coronal view); (C) thickness (anterior to posterior measurement, sagittal). (D–F) Free‐hand scripting segmentation procedure utilized in CT/MRI volumetric analyses to estimate differential parenchymal volume and split renal function. Transverse measurements of areas are made at 3 mm intervals and then summed to give a direct estimate of the parenchymal volumes in the ipsilateral versus the contralateral kidney, as described in Mir et al.^18^ (D) segmentation to obtain the parenchymal volume of the contralateral kidney. (E) Segmentation to obtain the total volume of the ipsilateral renal parenchyma and tumour. (F) Segmentation to obtain the volume of the tumour alone, which is then subtracted from (E) to determine the preoperative ipsilateral parenchymal volume. (G–I) Semi‐automated software‐derived parenchymal volume analysis (PVA) to estimate the SRF (Fujifilm medical systems) as described in Rathi et al.^6^ (G) Measurement of the volume of the contralateral kidney. (H) Volume of the ipsilateral kidney + tumour. (I) Volume of the tumour alone, which is then subtracted from (H to determine the ipsilateral parenchymal volume. SRF is based on the relative amounts of parenchyma on each side normalized by total parenchymal volume. (J–L) Use of semi‐automated software to determine PPVP following PN as described in Kazama et al.^19^ (J) Tumour plus parenchyma. (K) Tumour alone. (L) Parenchyma present in postoperative state. These techniques demonstrate the evolution of PVA calculation methods as each iteration improved the technique of determining SRF and PPVP. With all these methods it is important to exclude cysts, central sinus, collecting system and other unrelated structures when measuring the parenchymal volumes on each side before and after surgery.

**FIGURE 2:**
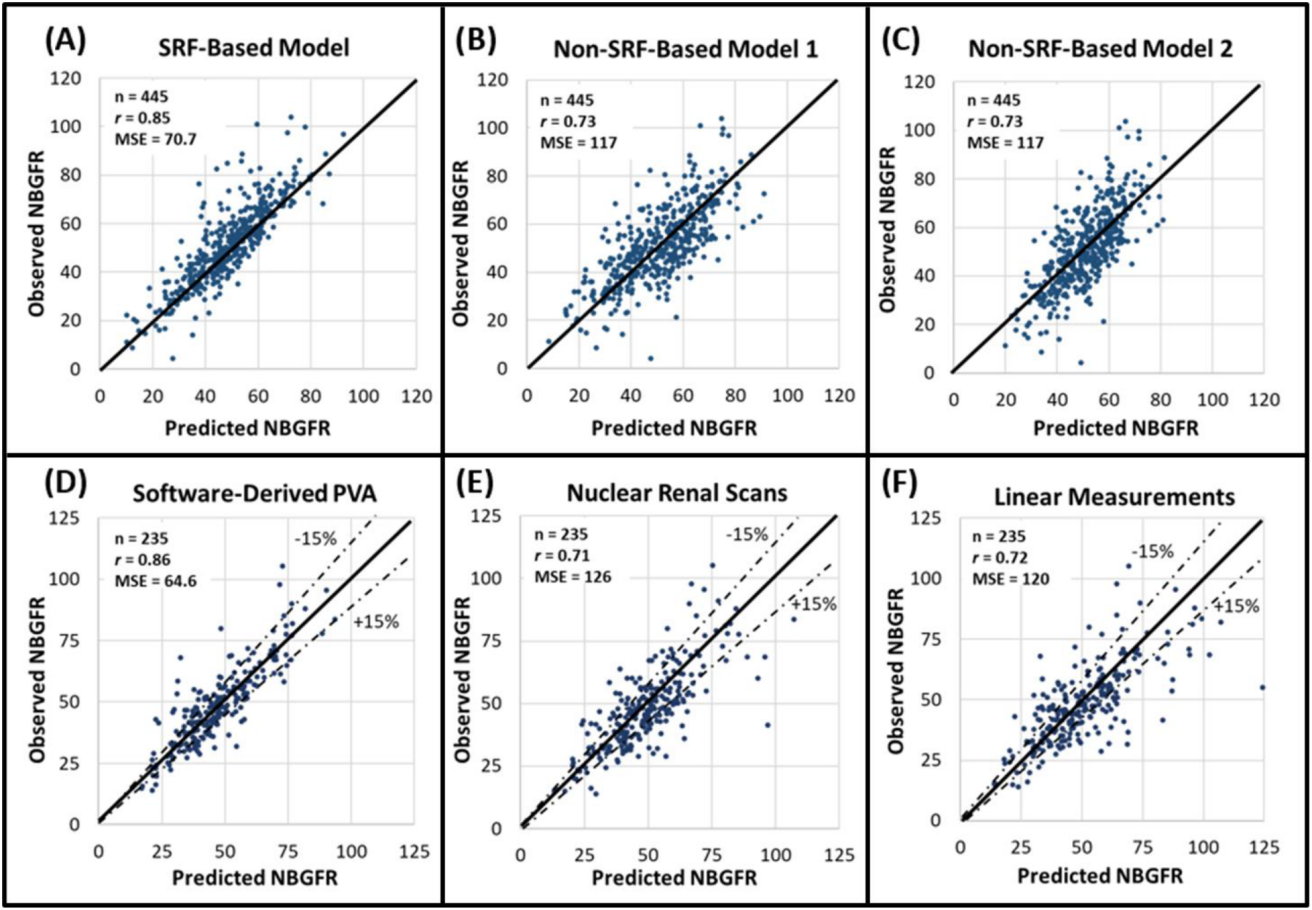
The utility of parenchymal volume analysis (PVA) for estimating split renal function (SRF) and predicting functional outcomes after radical nephrectomy (RN). (A–C) Show the accuracies of SRF‐ and non‐SRF‐based models for predicting new baseline GFR (NBGFR) after RN (Rathi et al.^5^). The SRF‐based model is *predicted NBGFR = 1.24 x preoperative GFR x SRF*_*Contralateral*_ (Rathi et al.^6^), with 1.24 representing the average amount of renal functional compensation observed in adults after RN. B) Non‐SRF‐based model 1 is *predicted NBGFR = 17 + preoperative GFR (×0.65) − age (×0.25) + 3 (if tumour>7 cm) − 2(if diabetes)* (Aguilar Palacios et al.^27^). (C) Non‐SRF‐based model 2 is *predicted NBGFR = 29.8–0.235 x age − 3.30 (if diabetes) + 0.457 x preoperative eGFR − 1.39 (if proteinuria) + 0.401 x size of renal mass (cm) + 0.148 x months post‐RN − 0.0020 x age x months post‐RN* (Bhindi et al.^22^). (D–F) Show the accuracies of different approaches for estimating SRF and, by extension, predicting NBGFR after RN (Rathi et al. *Sci Rep.* ^21^). (D) SRF was obtained from semi‐automated software‐derived parenchymal volume analysis. (E) SRF was obtained from Tc‐99 m MAG3 nuclear renal scans. (F) SRF was derived from PVA based on the product of linear measurements of the renal length, width and thickness. PVA from semi‐automated software estimates provides the most accurate estimates of SRF and NBGFR after RN (p values <0.05 when compared to the other methodologies used in E or F).

The simplest approach for PVA involves linear measurement of parenchymal dimensions (height/width/length), to estimate parenchymal volume (Figure 1A–C). This method has been utilized in both kidney transplant and cancer patients for the past few decades, with slight variations in technique^16^, ^17^ (Table 1). A similar approach presumes that the renal contour approximates a cylinder, and a simple measurement of the length and radius of the kidney will yield a reasonable estimate of the parenchymal volume^20^ (volume = length×πr^2^). Linear measurement‐based PVA estimates of SRF correlate closely with NRS‐based SRF estimations, and equivalence for predicting NBGFR after nephrectomy^21^ (Table 1, Figure 2F). This is the least sophisticated of the PVA methods, because linear measurements are subjective and fail to capture the complexity of a 3D organ, and endophytic tumours can complicate the measurements. These simple methods also have notable benefits, including ease‐of‐use, speed and accessibility, because specialized software is not required. Similar to all PVA approaches, they obviate the need for additional imaging studies, namely NRS, with its associated costs and exposure to a radioactive isotope.

**TABLE 1:** Studies evaluating split renal function derived from various approaches and correlations with renal functional outcomes.

Author & reference	PVA method(s) Used	Comparison group(s)	Primary functional outcome	Sample size and population	Conclusion	Predictive performance
Feder et al., *J Urol*. 2008 (PMID: 18804236)	Linear renal measurement	Tc‐99 m MAG3 scans	Correlation of PVA‐based split renal function estimation with NRS‐based estimation	111 patients who underwent CT and Tc‐99 m MAG3 (any indication)	Differential PVA by CT correlated with differential function on Tc‐99 m MAG3	*r* = 0.959 for correlation between PVA‐based vs Tc‐99 m MAG3‐based predictions of SRF. Suggests that renal scan not required.
Miyazaki et al., *Ann Nucl Med*. 2010 (PMID: 20213340)	Segmentation	DTPA/Planar; DTPA/SPECT; (99 m)Tc‐DMSA/SPECT	Correlation of PVA‐based split renal function, DTPA/Planar; DTPA/SPECT; and DMSA/SPECT (reference)	60 live kidney donors	SRF from DTPA/SPECT was more accurate than DTPA/P. PVA also provided accurate estimates of SRF.	*R values*: 0.66 for DTPA/P, 0.85 for DTPA/SPECT, and 0.91 for PVA. Volume measurements from CT were non‐inferior to renal scan‐based approaches.
Morrisroe et al., *J Urol*. 2010 (PMID: 20400144)	Segmentation	Tc‐99 m MAG3 scans	Correlation of PVA‐based split renal function estimation with NRS‐based estimation	33 patients with CT (10 without contrast, 23 with contrast) and Tc‐99 m MAG3	Differential PVA by CT correlated with differential function on Tc‐99 m MAG3.	*r* = 0.90 for predicted (PVA) vs observed (Tc‐99 m MAG3) renal function. *r* = 0.87 for enhanced CT, 0.95 for nonenhanced CT. Supports use of PVA, NRS may not be needed.
Kato et al., *Eur J Radiol*. 2011 (PMID: 19963330)	Software‐based (Fujifilm and Ziosoft)	Tc‐99 m DMSA scans	Correlation of PVA‐based split renal function estimation with NRS‐based estimation	28 living renal donors with CT and NRS	Fujifilm‐based PVA was as accurate as ZIOSOFT. PVA and NRS were concordant in identifying the dominant side.	R^2^ = 0.91 between Fujifilm‐PVA and Ziosoft‐PVA. R^2^ = 0.87 for Fujifilm‐SRF and NRS. NRS and Fujifilm‐SRF showed agreement regarding dominant side in 26 of 28 cases.
Ramaswamy et al., *Can J Urol*. 2013 (PMID: 23930608)	Linear renal measurement	Tc‐99 m MAG3 scans	Correlation of PVA‐based split renal function estimation with NRS‐based estimation	47 patients who underwent laparoscopic pyeloplasty for ureteropelvic junction obstruction and had both NRS and CT.	Differential PVA by CT correlated with differential renal function on NRS.	*r* was approximately 0.90 for predicted (PVA) vs observed (NRS). Suggests that NRS may not be necessary in some situations.
Diez et al., *Clin Transplant*. 2014 (PMID: 24654729)	Segmentation	Tc‐99 m MAG3 scans	Correlation of PVA‐based split renal function estimation with NRS‐based estimation	65 living kidney donors with available CT imaging and NRS.	Differential PVA by CT correlated with differential function on Tc‐99 m MAG3.	*r* = 0.59 for PVA‐based SRF and NRS‐based SRF.
Yanishi et al., *Transplant Proc*. 2015 (PMID: 26680075)	Software‐based (Fujifilm)	Tc‐99 m MAG3 scans	Correlation of PVA‐based split renal function estimation with NRS‐based estimation; NBGFR estimation (predicted vs observed)	35 living kidney donors who underwent nephrectomy with preoperative CT and NRS.	PVA from CT correlated with SRF from Tc‐99 m MAG3, and correlated with NBGFR in the donor.	*r* = 0.71 for PVA‐based SRF vs. NRS‐SRF. *r* = 0.71 for PVA‐NBGFR vs. observed. *r* = 0.63 for NRS‐NBGFR vs. observed. Suggests CT‐based measurements may suffice.
Wahba et al., *Transplantation*. 2016 (PMID: 26356175)	Linear renal measurement (ellipsoid); segmentation (parenchymal or cortical volume)	Tc‐99 m MAG3 scans	Correlation of PVA‐based split renal function estimation with NRS‐based estimation	101 living kidney donors with preoperative CT and NRS.	SRF from PVA correlated with SRF from NRS and with donor eGFR post‐donation.	*r* = 0.85–0.88 for PVA‐based SRF vs. observed eGFR at day 3 post‐transplant; versus 0.84 when NRS was used. *r* = 0.75–0.77 for PVA‐based SRF vs. observed eGFR at 1 year post‐transplant; 0.73 for NRS. Suggest NRS may not be needed.
Mitsui et al., *Clin Exp Nephrol*. 2018 (PMID: 28741049)	Software‐based (Fujifilm)	Tc‐99 m DMSA scans	Correlation of PVA‐based split renal function estimation with NRS‐based estimation	63 patients who underwent live donor nephrectomy.	Strong correlations between SRF from PVA and NRS. NBGFR after donation correlated with SRF measured by NRS and software‐based PVA.	*r* = 0.92 for software‐based PVA and SRF from NRS. Supports use of CT‐based measurements of differential parenchymal volumes to estimate SRF and functional outcomes in transplant donors.
Ye et al., *BJU Int*. 2020 (PMID: 31971315)	Segmentation; software‐based (Aquarius intuition)	Tc‐99 m MAG3 scans	Correlation of PVA‐based split renal function estimation with NRS‐based estimation; function saved vs parenchymal volume saved after PN	374 patients with renal cell carcinoma (RCC) who underwent PN, 155 patients with RCC who underwent RN.	PVA provides accurate estimations of SRF and can be evaluated using software analysis or freehand scripting.	*r* = 0.94 for PVA vs. NRS for preoperative ipsilateral eGFR. *Relationship between function saved and parenchymal volume saved was much sharper when SRF was derived from PVA rather than NRS (p < 0.05)*.
Schober et al., *BJU Int*. 2024 (PMID: 37667554)	Linear renal measurement (RENAL‐MS)	Tc‐99 m MAG3 scans	NBGFR estimation (predicted vs observed)	57 patients with abdominal CT/MRI and NRS prior to RN	RENAL‐MS is equally accurate as NRS for predicting SRF, NBGFR and postoperative Stage 3 chronic kidney disease (CKD) after RN.	*r* = 0.82 for RENAL‐MS, 0.76 for NRS. AUC (Stage 3 CKD) = 0.93 for RENAL‐MS, 0.97 for NRS. Suggests simple linear measurements from CT can estimate SRF as well as NRS.
Rathi et al., *Sci Rep*. 2023 (PMID: 37069196)	Software‐based (Fujifilm); linear renal measurement	Tc‐99 m MAG3 scans	NBGFR estimation (predicted vs observed)	235 patients with RCC managed with RN who had preoperative CT/MRI and NRS.	Software‐derived PVA provides the most accurate estimates of SRF and predictions of NBGFR after RN. The linear measurements approach is equally accurate as NRS.	*r* = 0.86 for observed vs. predicted NBGFR for software‐derived PVA, 0.71 for NRS, 0.72 for linear measurements. *Software‐derived PVA method was significantly better than the other 2 methods (p < 0.05). Confirms NRS no longer needed in many patients*.

AUC: Area Under the Curve; CT: Computed Tomography; DMSA: Dimercaptosuccinic Acid; DMSA/SPECT: Dimercaptosuccinic Acid with Single Photon Emission CT; DTPA: Diethylene Triamine Penta‐Acetic Acid; DTPA/P: DTPA with Planar Scintigraphy; DTPA/SPECT: DTPA with Single Photon Emission CT; eGFR: Estimated Glomerular Filtration Rate; MELV: Modified Ellipsoid Volume; MRI: Magnetic Resonance Imaging; NRS: Nuclear Renal Scans; PVA: Parenchymal Volume Analysis; *r*: correlation coefficient; RCC: Renal Cell Carcinoma; RCV: Renal Cortex Volume; RENAL‐MS: Real‐Time Estimation of Nephron Activity with a Linear Measurement System; ROI: Region of Interest; SRF: Split Renal Function; SRV: Split Renal Volume; Tc‐99 m MAG3: Technetium‐99 Mercaptoacetyltriglycine; 99 m Tc‐DMSA: Technetium‐99 Dimercaptosuccinic Acid.

#### 2.3.2 Segmentation analysis

Segmentation analysis addresses some of these disadvantages by segmenting the kidney on cross‐sectional imaging to directly measure the parenchymal volumes (Figure 1D–F). Using CT or MRI, the areas of axial slices (3–5 mm thickness) are measured using free‐hand scripting. These areas are multiplied by slice thickness and summed to yield the parenchymal volume. Non‐parenchymal tissue (e.g. renal sinus/vessels/tumours) is excluded from the measurements. Segmentation‐based PVA correlates closely with SRF derived from NRS across various patient groups, including renal transplant, obstructive uropathy and kidney cancer^18^, ^23^ (Table 1). Segmentation is more precise than linear renal measurements because it accounts for renal contour complexity while excluding non‐parenchymal structures. However, it is time‐consuming and there is still some element of subjectivity with segmentation, although to a lesser degree than the linear measurement method.

#### 2.3.3 Automated software analysis

With the rise of artificial intelligence and other automated image‐processing platforms, there has been rapid growth in 3D volumetric analyses. These new technologies have been applied to PVA, leading to significant improvements in accuracy, reproducibility and processing time. They rely on cross‐sectional imaging such as CT and MRI and automatically identify the density and contours of the renal parenchyma to perform volumetric measurements (Figure 1G–I). These advanced software applications can identify and exclude extra‐parenchymal structures like the renal sinus/vessels/tumours/cysts in an automated fashion with minimal human manipulation and are therefore more objective. Most applications require minimal training and typically can be performed in just a few minutes.

Software‐based automated PVA for estimating SRF was first applied for living‐donor transplant candidates. In most donors, the left kidney is preferred for donation due to its longer renal vein, however, if it is contributing a significantly disproportionate SRF to the global GFR, the right kidney will be removed instead. Across multiple studies, software‐based differential PVA has proven equivalent or superior to NRS for the estimation of SRF^24^, ^25^, ^26^ (Table 1), and has essentially replaced NRS for the evaluation of potential transplant donors at most centres. Some studies have demonstrated that volumetric analysis specific to the renal cortex, rather than the entire parenchyma as in standard PVA, might improve SRF estimates.^27^ However accurate discrimination of the renal cortex from the medulla can be challenging depending on the quality of the imaging, and this is not a practical approach for most patients.

Moving forward from this experience with renal transplantation, there was then a natural interest in leveraging PVA software to estimate SRF in patients under consideration for RN for kidney cancer. However, there were concerns about whether PVA could be used in this population, which is older and more likely to have renal comorbidities that could affect the parenchymal volume/function relationship. Other concerns were that the tumour could complicate the measurements of parenchymal volumes, as could other anatomic abnormalities that might be more prevalent in the elderly (e.g. renal vascular disease or prior renal surgery).

The potential application of PVA in kidney cancer was evaluated by Ye,^28^ who found that automated PVA‐based SRF estimations correlated strongly with NRS‐based estimations in both PN and RN patients, assuaging concerns that PVA would not be feasible in this population. Subsequent studies by Rathi further built upon these findings, demonstrating that software‐based automated PVA was significantly more accurate than NRS at predicting NBGFR following RN.^21^ For example, Rathi reported that the correlation between observed and predicted NBGFR after RN was significantly stronger when PVA was used to determine SRF compared to linear measurements or NRS^21^ (Figure 2, *r =* 0.86/0.72/0.71, respectively, p < 0.05). PVA‐based prediction of NBGFR after RN was also superior to various non‐SRF‐based algorithms^5^, ^29^ (Figure 2).

Further support for the superiority of software‐based PVA comes from studies evaluating outcomes for patients managed with PN. The literature has demonstrated that the percent‐parenchymal‐volume‐preserved (PPVP) in the ipsilateral kidney correlates strongly with GFR preserved in that kidney.^28^, ^30^ Such studies require estimation of SRF to assess the functional recovery specific to the kidney exposed to ischemia. These studies have shown that the correlation between PPVP and ipsilateral GFR saved is strongest when software‐based PVA methods are used to determine SRF, suggesting their improved accuracy compared to alternative methods for SRF estimation such as NRS.

### 2.4 Prediction of new baseline GFR after PN

Prediction of NBGFR after PN can also be important for patient counselling. Several multifactorial algorithms have been developed from large databases that provide modest accuracy. However, previous studies have supported the 80/90 rule, which states that the average amount of function saved in the operated kidney after PN is 80%, and if there is a contralateral kidney, the amount of global function saved averages 90%.^4^ Strong anchoring by the preoperative GFR and minimal functional loss with the typical PN likely account for these observations. Based on this, one could simply estimate that the NBGFR after PN for most patients will be 0.9 × (preoperative global GFR). This simple approach has proven equivalent or better than all other published algorithms.^31^, ^32^

### 2.5 Limitations of PVA for estimation of NBGFR after nephrectomy

As the role of PVA has expanded, concerns have been raised regarding potential limitations. These primarily centre around the validity of the central assumption that renal parenchymal volume directly correlates with function. This issue is a concern in kidney cancer patients, who tend to be older and have added complexities in determining parenchymal volume due to the presence of malignancy.

A recent study explored patient and tumour‐related factors that may correlate with inaccuracies of PVA‐based estimation of NBGFR following RN.^33^ The data suggested that patient comorbidities such as cardiac disease and obesity did not impact the accuracy of PVA, likely because these are systemic processes that impact both kidneys equally. However, advanced age correlated with significant overprediction of NBGFR, likely related to reduced RFC in this population rather than inaccuracies in PVA itself. Certain factors that disrupt the volume‐function relationship of the kidney, such as hydronephrosis, are associated with PVA inaccuracy. For instance, the use of PVA in patients with hydronephrosis in the tumour‐bearing kidney will typically lead to underprediction of the NBGFR after RN. In this setting, the ipsilateral parenchyma is not functioning optimally, and the global GFR is mostly derived from the contralateral kidney, so the NBGFR will be higher than PVA would predict.^33^

Additionally, factors that make accurate discrimination of renal parenchyma challenging, such as infiltrative renal masses or predominance of renal cysts, also correlated with PVA inaccuracy. Importantly, even in cohorts where PVA was less accurate, which represented 22% of the overall cohort, it was still non‐inferior to NRS, suggesting it may be a suitable option even in that setting.

### 2.6 Other clinical applications of PVA

Another emerging application of PVA in urology is percutaneous‐nephrolithotomy (PCNL) for renal stones, which could induce renal atrophy. Recent PVA studies have shown that a history of multiple PCNLs and higher stone burden is associated with more pronounced atrophy, and efforts to minimize this are now under study.^34^ PVA has also been used in urologic oncology to study parenchymal‐volume‐replacement (PVR), which reflects the discordant loss of healthy parenchyma due to invasive tumour growth. As renal tumours grow, they can displace parenchyma or obliterate it in an aggressive manner. Recent studies have demonstrated that increased PVR is associated with decreased preoperative ipsilateral GFR and adverse pathology.^35^, ^36^ Renal volumetric assessments have also shown promise in paediatric populations, including assessing pyeloplasty outcomes in ureteropelvic‐junction‐obstruction and quantifying parenchymal volume loss in reflux‐nephropathy.^37^, ^38^ These studies have generally utilized ultrasound rather than cross‐sectional imaging, and the accuracy of ultrasound‐based parenchymal measurements remains less extensively validated.

### 2.7 Future directions

At present, the best prediction model following RNU comes from Hensley, who utilized various preoperative factors to develop a predictive nomogram.^39^ However, this model, which did not incorporate SRF, only provided modest accuracy for predicting NBGFR (*r* = 0.66). PVA has not been studied in patients with UTUC due to concerns about the increased incidence of ureteral obstruction and infiltrative growth patterns, which are associated with PVA inaccuracy. These concerns remain theoretical, and further research is needed to determine PVA's efficacy in this patient population. Incorporation of software‐based assessment of the degree of parenchymal enhancement along with parenchymal volume measurements may improve the prediction of SRF, and this is currently under investigation.

Much of the recent advances in PVA have been driven by artificial intelligence, which utilizes deep learning models to perform automated segmentation of renal parenchyma and detection of renal masses.^40^ This promising technology continues to increase the accuracy and throughput of PVA and provides the backbone for much of the commercially available software for volumetric studies.

## 3 RESEARCH APPLICATIONS

### 3.1 PVA for study of functional recovery after PN

PN has traditionally been performed with vascular occlusion to provide a bloodless field for tumour resection and reconstruction of the kidney.^4^ However, this exposes the kidney to ischemia, which can deleteriously affect functional recovery.^4^ The impact of ischemia during PN has been very contentious in the field of localized kidney cancer. This controversy has evolved through several paradigms over the past decade reflecting the tools available at the time, and conceptual advances that have occurred (Table 2).

**TABLE 2:** Evaluating determinants of functional recovery after partial nephrectomy in different eras.

Era	Inclusion of parenchymal volume analysis (PVA)	Summary of key findings and interpretations	Strengths and limitations of PVA technique	Relevant References
Prior to incorporation of percent parenchymal volume preserved (PPVP, pre‐2010)	No	The primary determinants of NBGFR after PN appeared to be preoperative eGFR, presence of a solitary kidney, age, gender, tumour size and ischemia time. Duration of ischemia appeared to be the strongest modifiable risk factor for decreased NBGFR. Longer duration of warm ischemia correlated with increased risk of acute renal failure and de novo stage IV CKD.	Not applicable: It was not recognized that the PPVP was important, so it was not estimated or measured *“Every minute counts”* was the conclusion, which subsequently has been shown to be misleading, it was a correlation not cause‐effect, and ischemia was mostly a confounder.	Lane et al., *J Urol*. 2008 (PMID: 18930264) Thompson et al., *Eur Urol*. 2010 (PMID: 20825756)
Subjective Estimation of PPVP (2011–2012)	Yes: Subjective estimation by the operating surgeon	On multivariable analysis, preoperative eGFR and estimated percent of parenchyma spared were the primary determinants of NBGFR and new‐onset CKD. *Quality and Quantity predominated*	Subjective estimation Strengths: quick and easy, and minimal resources needed. Limitations: reduced accuracy and not as reproducible. *Main take‐home point: When PPVP was incorporated, ischemia lost statistical significance*	Lane et al., *J Urol*. 2011 (PMID: 21167524) Thompson et al., *Urology*. 2012 (PMID: 22310752)
Quantitative Approaches (2013–2019)	Yes Manual cross‐sectional segmentation (free‐hand scripting) of CT/MRI scans Cylindrical volume approximation Nuclear renal scans used for SRF	Percent GFR preserved is associated with % renal parenchyma preserved, lower R.E.N.A.L. scores, and the use of cold ischemia. Recovery from ischemia was 90–100% (1–12 months after PN), even for poorly functioning kidneys. However, duration of ischemia can contribute to acute changes in GFR. Devascularized parenchymal mass has a greater impact than excised parenchymal mass on functional recovery after PN. The average percent parenchymal volume preserved and function is 80% in the ipsilateral kidney. From a global standpoint, on average PN saves about 90% of the preoperative function for patients with two kidneys.	Manual cross‐sectional segmentation (free‐hand scripting) of CT/MRI Strengths: Accurate estimates of parenchymal volumes. Limitations: Labour‐intensive, time‐consuming and high inter‐rater variability. Cylindrical volume approximation Strengths: quick and easy Limitations: Questionable accuracy and reproducibility due to subjectivity. Nuclear renal scans Strengths: “traditional reference standard” for estimating SRF. Limitations: requires extra imaging with radiotracer exposure and tends to be less accurate than PVA.	Mir et al., *Urology* 2013 (PMID: 23791213) Ginzburg et al., *Urology*. 2015 (PMID: 26199171) Zargar et al., *BJU Int*. 2015 (PMID: 24905965) Zhang et al., *Eur Urol*. 2016 (PMID: 26525838) Dong et al., *J Urol*. 2017 (PMID: 28400188) Dong et al., *Eur Urol Focus*. 2018 (PMID: 28753855) Isharwal et al., *J Urol*. 2018 (PMID: 29225058)
Software‐derived PVA (2015–2024)	Yes: Software‐derived PVA	Accurate, objective and reproducible PVA allows for more precise analyses of the effects of clinical, tumour and surgical parameters that may affect NBGFR after PN. Parenchyma preserved is the primary determinant of functional recovery after PN. Renal comorbidities, age, warm ischemia and ipsilateral parenchymal atrophy predict longitudinal ipsilateral functional decline. PVA allows for a more discerning analysis of secondary factors affecting functional recovery after PN, beyond PPVP.	Software‐derived PVA Strengths: Software is quick, user‐friendly and can be applied at point of care in clinical settings; provides objective and reproducible estimates of renal parenchymal volume; and no extra imaging/lab studies are needed. *PVA allows for more accurate analysis of percent parenchymal volume preserved (PPVP) and more accurate estimation of SRF. It also facilitates the analysis of more robust populations*. Limitations: Accuracy may be compromised in cases of pyelonephritis, hydronephrosis, renal vein thrombosis or infiltrative tumours. After PN with prolonged warm ischemia, the parenchymal volume/function relationship may be disrupted, and PVA may overestimate functional recovery.	Mibu et al., *World J Urol*. 2015 (PMID: 25555568) Munoz‐Lopez et al., *BJU Int*. 2023 (PMID: 37017637) Munoz‐Lopez et al., *BJU Int*. 2023 (PMID: 37409822) Attawettayanon et al., *Urol Oncol*. 2024 (PMID: 38142208) Kazama et al., *BJU Int*. 2024 (PMID: 38355293)

CKD: Chronic Kidney Disease; CT: Computed Tomography; eGFR: estimated Glomerular Filtration Rate; MRI: Magnetic Resonance Imaging; NBGFR: New Baseline GFR; PN: Partial Nephrectomy; PPVP: Percent Parenchymal Volume Preserved; PVA: Parenchymal Volume Analysis; R.E.N.A.L.: Radius, Exophytic/Endophytic Location, Nearness to the Collecting System, Anterior or Posterior Location, Location Relative to the Renal Poles; SRF: Split Renal Function.

In 2010, our multicentre group published ‘Every minute counts when the renal hilum is clamped during PN’.^41^ Each additional minute of warm ischemia during PN correlated with a 5% increased risk of AKI and 6% increased risk of new‐onset stage‐4 CKD. Our group was not the first to study this, and other groups reported similar results.^42^, ^43^ As such, ischemia was considered the primary determinant of functional recovery following PN, which galvanized efforts to minimize ischemia through segmental clamping or off‐clamp approaches.

Although these data garnered significant attention, they told an inaccurate story. After publishing ‘Every minute counts’ our multicentre collaborative studied a more robust cohort of patients with renal mass in a solitary kidney, managed with either warm ischemia or hypothermia.^44^, ^45^ The initial data were analogous – every minute counted. However, we wondered if the percent‐parenchymal‐volume‐preserved (PPVP) would also affect functional outcomes. When PPVP was incorporated into the analysis, it was the primary predictor and ischemia lost statistical significance. Importantly, in this study, the data regarding PPVP was based on subjective estimation by the surgeon rather than objective measurement, yet it still proved to be of primary importance. Based on this, we concluded that PPVP, rather than ischemia, was the primary determinant of functional outcomes following PN. As such, our group began to study functional outcomes by focusing on the outcome parameter Rec_ischemia_, defined as the percent ipsilateral GFR saved normalized by PPVP, which would be 100% if all preserved nephrons recovered completely from exposure to ischemia.^4^, ^18^, ^46^, ^47^ However, the technology to evaluate both PPVP and Rec_ischemia_ was destined to undergo substantial evolution in the subsequent years (Table 2).

Prior studies in this domain relied on the availability of NRS both pre‐ and post‐operatively to determine functional recovery specific to the operated kidney that was exposed to ischemia.^18^, ^48^, ^49^ Given these limitations, only 10–20% of patients within a given era could be analysed, raising concerns about ascertainment biases. The introduction of PVA offered a solution to this problem because most patients have pre‐ and postoperative cross‐sectional imaging available, which is all that is required for this approach. Additionally, the latest generation of studies has leveraged software‐based PVA to provide more accurate and objective estimations of SRF and PPVP.

### 3.2 Research Findings from PVA and clinical implications

We recently applied software‐based PVA to define PPVP and SRF more accurately and allow for a more discerning evaluation of secondary factors that may negatively impact functional recovery after PN.^30^ Six‐hundred and seventy PN patients were analysed, and the median loss of function was 7.8 ml/min/1.73m^2^, which was mostly (81%) due to loss of parenchymal volume. Independent predictors of functional recovery, in addition to PPVP, included renal‐related comorbidities (insulin‐dependent diabetes and refractory hypertension) and warm ischemia. Such secondary factors were not appreciated in prior studies due to the rudimentary nature of the available methodology.^50^ In this ‘next generation’ study, the relationship between ipsilateral GFR preserved and PPVP was stronger than ever observed (*r =* 0.83, p < 0.01), reflecting improved accuracy of the tools used in this study. Although these data were robust, they left other unanswered questions.^4^ Could PVA be used to study renal atrophy and long‐term functional decline after PN, and does exposure to ischemia leave the kidney vulnerable to such processes?

During the ageing process, the average adult loses renal function at a rate of 0.8 ml/min/1.73 m^2^ per year, or ~0.4 ml/min/1.73 m^2^ per kidney per year, in part due to minimal but progressive atrophy. Munoz‐Lopez studied a population of 349 patients with a longitudinal follow‐up that would allow for the estimation of ipsilateral parenchymal atrophy and longitudinal functional decline.^51^ A main finding was that the average PN patient experienced longitudinal functional decline in line with natural aging, despite exposure to ischemia and no difference was observed between the ipsilateral and contralateral kidneys. When patients were stratified by comorbidity status, the authors found that patients with significant renal comorbidities (refractory hypertension, diabetes with insulin‐dependence or end‐organ damage or CKD stage ≥IV) experienced 2–3 fold increased rates of functional decline and longitudinal atrophy beyond what would be expected by ageing, and again this was seen equally in both kidneys. The authors concluded that the primary determinants of longitudinal functional decline are patient comorbidities, rather than prior exposure to ischemia. PVA helped facilitate this study by providing precise estimates of parenchymal volumes, allowing for a discerning analysis of progressive atrophy and functional decline.

These two studies also confirmed the primary importance of PPVP in determining functional outcomes after PN, both short and long‐term. However, their focus was on secondary factors rather than PPVP. In 2024, Kazama studied a robust population of PN patients (n = 894) and sought to more rigorously explore factors associated with PPVP.^19^ Increased tumour complexity is associated with reduced PPVP, while the presence of a solitary kidney and use of cold ischemia are associated with increased PPVP. The association between hypothermia and improved PPVP may relate to the surgeon feeling they are ‘off the clock’ and therefore able to minimize PPVP by selectively utilizing tumour‐enucleation, optimizing placement of intraparenchymal sutures and carefully closing the capsule to minimize devascularization. Improved PPVP for solitary kidneys likely reflects recognition of the importance of preservation of renal parenchyma in this imperative setting. These studies have strengthened our understanding of the factors influencing PPVP and were facilitated by PVA and its improved accuracy for measuring parenchymal volumes. However, these PN studies had a predominance of cases with limited ischemia times (<25 minutes), leaving knowledge gaps regarding the potential deleterious impact of prolonged ischemia.

Renal masses with high complexity are challenging and may require extended clamp times to achieve complete resection and safe reconstruction. For patients with a solitary kidney or preexisting CKD, this may negatively affect long‐term functional outcomes, potentially leading to an increased risk of dialysis dependence. In this setting, there are several controversies, including the protective role of hypothermia and the threshold of warm ischemia that is associated with irreversible ischemic damage. A recent study evaluated 197 PN cases with prolonged ischemia >30 minutes (88 warm and 109 cold ischemia).^52^ The results indicate that Rec_ishemia_ begins to decline after 30 minutes of warm ischemia in a statistically significant manner, with each additional 10‐minute interval associating with 4% further decline in functional recovery. Similar deleterious effects were not seen in the prolonged cold ischemia cohort, supporting a protective effect of hypothermia. However, functional recovery in the setting of prolonged ischemia can be highly variable, with some patients displaying surprisingly positive functional outcomes even after rather extended periods of warm ischemia.^53^ Further study will be necessary to explore this important topic.

### 3.3 Limitations of PVA for analysing functional recovery after PN

Despite the promise of PVA for accurate estimation of functional outcomes, there are concerns regarding potential limitations, particularly post‐PN. It has been hypothesized that ischemia during clamped PN may irreversibly damage the ipsilateral renal parenchyma, and thus disrupt the volume/function relationship that PVA depends on for accurate SRF estimates. If this occurs, PVA would overestimate the SRF of the kidney exposed to ischemia, and thus overestimate the functional recovery of that kidney. This is currently under investigation in patients who have undergone both PVA and NRS post‐PN (n = 437). Preliminary data suggests that PVA overestimation may occur to a modest degree in patients managed with prolonged warm ischemia (>25 minutes). In these patients, discordance was observed between the median ipsilateral SRF obtained from PVA versus NRS (42 vs 39%, respectively, p < 0.05). The degree of PVA/NRS discordance increased with longer warm ischemia times, suggesting a modest degree of progressive irreversible damage to the ipsilateral kidney, confirming the main findings of Kazama.^52^ When a correction factor for PVA overestimation was applied to Kazama's data,^52^ the degree of decline of ipsilateral function with each additional 10 minutes beyond 30 minutes was ≈9%, about 2‐fold higher than estimated by PVA analysis alone. In contrast, PVA/NRS discordance was not observed for limited warm ischemia (<25 minutes) or hypothermia of any duration, suggesting that PVA remains highly accurate for the estimation of SRF and functional outcomes after PN in the great majority of cases.

## 4 CONCLUSIONS

PVA offers an inexpensive, readily available and highly reproducible method of estimating SRF and parenchymal volumes in patients with renal tumours and can guide decision‐making for renal transplantation. PVA‐derived SRF‐based models provide more accurate predictions of NBGFR after RN compared to other nomograms or NRS‐based estimations. Moreover, software‐based PVA has enabled a more discerning analysis of secondary factors affecting functional recovery following PN, which can impact the surgical approach. PVA is ready for prime‐time implementation in these domains and holds significant promise for further advances in patient care and research endeavours. Despite the wide availability of PVA, primarily for use in renal transplantation, it has not been used for other urologic purposes at most centres. Our hope is that this narrative review will increase PVA utilization in urology and lead to further progress in the field.

## AUTHOR CONTRIBUTIONS

*Manuscript writing*: Carlos Munoz‐Lopez, Kieran Lewis, Nityam Rathi, Eran Maina, Akira Kazama, Anne Wong, Akira Kazama, Christopher Weight and Steven C. Campbell. *Data collection*: Angelica Bartholomew, Carlos Munoz‐Lopez, Kieran Lewis, Nityam Rathi and Eran Maina. *Supervision*: Worapat Attawettayanon, Yunlin Ye, Zhiling Zhang, Wen Dong, Rebecca A. Campbell, Nicholas Heller, Erick Remer, Christopher Weight and Steven C. Campbell.

## CONFLICT OF INTEREST STATEMENT

The authors declare no conflicts of interest.

## Supporting information


**Table S1:** Models and algorithms for predicting functional outcomes after partial and/or radical nephrectomy.
