# Supplementary material for: Renal parenchymal volume analysis: Clinical and research applications
Source: BJUI Compass. 2025 Mar 19;6(3):e70013. doi: 10.1002/bco2.70013 (PMC11922601; doi:10.1002/bco2.70013)
Supplement: Supplementary file 1 — Table S1: Models and algorithms for predicting functional outcomes after partial and/or radical nephrectomy. [file BCO2-6-e70013-s001.docx]

**Supplemental Table 1: Models and algorithms for predicting functional outcomes after partial and/or radical nephrectomy.**

| **Reference & Year** | **Cohort Size** | **Surgical**  **Intervention** | **Functional Outcome** | | **Timing after Surgery** | | **Predictive Variables** | **Inclusion of a Volumetric Parameter?** | **Predictive Performance** |
| --- | --- | --- | --- | --- | --- | --- | --- | --- | --- |
| Sorbellini et al., *J Urol.* 2006 (PMID: 16813869) | 1018  (161 PN and 857 RN) | PN/RN | Probability of postoperative serum creatinine > 2 mg/dl | | 7 years | | Preop serum creatinine, ASA score, % change kidney volume after surgery, patient age and sex | % change in kidney volume, PVA based on ellipsoid formula | Concordance Index: 0.83 |
| Kim et al., *J Urol*. 2009 (PMID: 19371883) | 359 (129 PN and 230 RN) | PN/RN | NBGFR | | 3 months | | PN: age, preop. creatinine clearance, and tumor size  RN: age, preop creatinine clearance, and weight | None | PN: R^2^ = 0.71  RN: R^2^ = 0.56 |
| Ohno et al., *J Urol.* 2011 (PMID: 21849191) | 271 | RN | New onset renal insufficiency (eGFR < 60) | | N/A | | Tumor size, patient age, preop eGFR | None | AUC: 0.92 |
| Yokoyama et al., *Int J Urol.* 2014 (PMID: 24118492) | 209 | RN | NBGFR, risk of postoperative CKD | | 3-4 years | | Age, presence of diabetes, preop eGFR | None | AUC: 0.79  R^2^ = 0.40 |
| Liss et al., *J Endourol.* 2016 (PMID: 26192380) | 130 (79 PN, 51 RN) | PN/RN | NBGFR,  renal insufficiency (eGFR < 60) | | 6 months | | Age and gender, ipsilateral and contralateral renal volumes, diabetes, hypertension, surgery type (PN vs RN), tumor size | Ipsilateral and contralateral kidney volumes from software-derived PVA | RMSE: 15.39  AUC: 0.89 |
| Shimada et al., *Investig Clin Urol*. 2017 (PMID: 28681031) | 422 | RN | NBGFR | | 1 year | | Males: Age, BMI, preoperative eGFR, tumor size  Females: age, preoperative eGFR | None | Males: R^2^ = 0.63  Females: R^2^ = 0.31 |
| Shum et al., *J Endourol.* 2017 (PMID: 28443676) | 461 | PN | NBGFR | | 1 year | | Age, race, sex, BMI, diabetes, hypertension, ischemic heart disease, stroke, preop creatinine, preop ipsilateral renal volume, presence of solitary kidney, tumor size | Ipsilateral kidney volume as assessed by PVA | R^2^ = 0.70 |
| Martini et al., *Eur Urol.* 2018 (PMID: 30224195) | 999 | PN | > 25% reduction in eGFR | | 3 – 15 months | | Age, sex, Charlson comorbidity index, preop eGFR, R.E.N.A.L. score | None | Concordance index: 0.73 |
| Raheem et al., *Int J Urol.* 2018 (PMID: 29923226) | 698 | PN | CKD-free survival | | 5 years | | Age, sex, diabetes, tumor size, and preop eGFR | None | Concordance index: 0.85 |
| Bertolo et al., *Eur Urol Oncol.* 2019 (PMID: 30929839) | 1897 | PN | Upstaging of CKD | | 3 months | | Age, sex, BMI, preop eGFR, R.E.N.A.L. score, ischemia time | None | AUC: 0.76 |
| Bhindi et al., *Eur Urol.* 2019 (PMID: 30477983) | 3072 (1920 PN, 1152 RN) | PN/RN | NBGFR | | 1 month | | PN: age, presence of a solitary kidney, diabetes, hypertension, proteinuria, preop eGFR, surgical approach, time since surgery | None | PN: R^2^ = 0.62 |
|  |  |  |  | |  | | RN: age, diabetes, proteinuria, preop eGFR, tumor size, time since surgery | None | RN: R^2^ = 0.41 |
| McIntosh et al., *BJU Int.* 2019 (PMID: 31145523) | 668 | RN | NBGFR < 45 | | 1 year | | Age, sex, preop creatinine | None | AUC: 0.78 |
| Shinoda et al., *Transpl Proc.* 2019 (PMID: 31076152) | 101 | Donor nephrectomy | NBGFR | | 1 year | | Age, BMI, preop eGFR, body surface area-adjusted preserved kidney volume | PVA | *r* = 0.80 |
| Aguilar Palacios et al., *J Urol.* 2020 (PMID: 32073996) | 273 | RN | NBGFR | | 3 – 12 months | | Age, preop eGFR, split renal function of contralateral kidney | Nuclear Renal Scan or PVA | *r* = 0.82 |
| Ellis et al., *J Am Soc Nephrol.* 2020 (PMID: 32238473) | 699 (187 PN, 512 RN) | PN/RN | NBGFR < 45 | 1 year | | Age, diabetes, preoperative eGFR, surgery type (PN or RN) | | None | Concordance Index: 0.84 |
| Karabay et al., *Int J Clin Pract.* 2021 (PMID: 33064933) | 154 | PN | NBGFR | 6 months | | Preop eGFR, diabetes, hypertension, BMI, surgery type, warm ischemia time, tumor size, tumor location, ASA score | | None | R^2^ = 0.90 |
| Aguilar Palacios et al., *J Urol.* 2021 (PMID: 33356481) | 7860 (3097 PN, 4763 RN) | PN/RN | NBGFR | 3 – 12 months | | Age, diabetes, preop eGFR, surgery type (PN or RN), tumor size | | None | R^2^ = 0.66 |
| Mari et al., *Eur Urol Focus.* 2022 (PMID: 34561199) | 981 | PN | > 25% reduction in eGFR | 48 months | | Age, sex, diabetes, Charlson comorbidity index, peripheral vascular disease, preop eGFR, surgical indication (elective vs relative vs imperative), PADUA score | | None | AUC: 0.82 |
| Wenzel et al., *Scand J Urol.* 2021 (PMID: 34427540) | 195 (129 PN, 66 RN) | PN/RN | Postoperative AKI (eGFR < 60) | 14 months | | Age, diabetes, hypertension, surgical type (PN or RN) | | None | N/A |
| Rathi et al., *World J Urol.* 2022 (PMID: 35022828) | 236 | RN | NBGFR,  NBGFR > 45 | 3 – 12 months | | Preop eGFR, split renal function from PVA, uniform measure for compensatory hypertrophy in adults (1.24) | | Software-derived PVA | *r* = 0.87  AUC = 0.94 |
| Rathi et al., *Ann Surg Oncol.* 2024 (PMID: 38006535) | 631 | PN | NBGFR | 3 – 12 months | | Preop eGFR, uniform measure for parenchymal volume preservation with the typical PN (0.90) | | None | *r* = 0.93 |
| Hensley et al., *Eur Urol Oncol.* 2024 (PMID: 38307832) | 1100 | RNU | NBGFR  Accuracy defined as predicted NBGFR within 30% of observed | 1 – 3 months | | Age, diabetes, hypertension, tumor size, preop eGFR | | None | *r* = 0.66  Accuracy: 78.6% |

AUC: Area Under the Curve; BMI: Body Mass Index; ASA: American Society of Anesthesiologists; CKD: Chronic Kidney Disease; eGFR: Estimated Glomerular Filtration Rate; NBGFR: New Baseline Glomerular Filtration Rate; PADUA: Preoperative Aspects and Dimensions Used for an Anatomical Nephrometry Score; PVA: Parenchymal Volume Analysis; *r*: correlation coefficient; R^2^: coefficient of determination; R.E.N.A.L.: Radius, Exophytic/Endophytic Location, Nearness to the Collecting System, Anterior or Posterior Location, Location Relative to the Renal Poles; RMSE: Root Mean Squared Error; RN: Radical Nephrectomy; RNU: Radical Nephroureterectomy
